# New inroads into the brain circuits and network dynamics behind sudden unexpected death in epilepsy

**DOI:** 10.1093/braincomms/fcac097

**Published:** 2022-04-13

**Authors:** Alfredo Gonzalez-Sulser

**Affiliations:** Simons Initiative for the Developing Brain, Patrick Wild Centre, Centre for Discovery Brain Sciences, University of Edinburgh, Edinburgh EH8 9XD, UK

## Abstract

This scientific commentary refers to ‘Hyperexcitable superior colliculus and fatal brainstem spreading depolarization in a model of sudden unexpected death in epilepsy’ by Cain *et al*. (https://doi.org/10.1093/braincomms/fcac006) and ‘Ictal neural oscillatory alterations precede sudden unexpected death in epilepsy’ by Gu *et al*. (https://doi.org/10.1093/braincomms/fcac073)

**This scientific commentary refers to ‘Hyperexcitable superior colliculus and fatal brainstem spreading depolarization in a model of sudden unexpected death in epilepsy’ by Cain *et al*. (https://doi.org/10.1093/braincomms/fcac006) and ‘Ictal neural oscillatory alterations precede sudden unexpected death in epilepsy’ by Gu *et al*. (https://doi.org/10.1093/braincomms/fcac073)**

Sudden unexpected death in epilepsy (SUDEP) is a devastating occurrence as patients perish prematurely and without warning. Despite SUDEP being the second leading cause of death in neurological disorders,^1^ the underlying mechanisms leading to SUDEP are still unknown and we are limited to merely identifying SUDEP risk factors, such as the frequency of occurrence of generalized tonic-clonic seizures and chronic uncontrolled epilepsy.^2^ In this issue of Brain Communications, two studies in rodent models provide new insights into the events leading to SUDEP: Cain *et al*., identify the superior colliculus as a potential key mediator of spreading depolarization (SD) propagation to the brain stem, which is thought to be the main mechanism leading to cardiac and respiratory arrest in SUDEP.^3^ While Gu *et al*.^4^ find suppressed brainstem oscillations, changes in cortical phase-amplitude coupling and a high degree of synchronous activity between the cortex and the brainstem preceding seizures that lead to sudden death.

SD, which is also known as spreading depression, is a slow and intense neuronal and glial depolarization wave in the grey matter of the central nervous system, which is accompanied by swelling of cells and distortion of dendritic spines.^5^ Transmembrane ionic gradients are broken down during these events, making action potential firing and neuronal activity nearly impossible. Seizures frequently occur in conjunction with SD. The precise relationship between the two is hotly debated as seizures can both precede and follow SD, while they can also occur independently from one another. Some modelling studies suggest that seizures and SD form part of a continuum of possible membrane dynamic states.^6,7^

Recent work in rodent models has shown that an important component leading to SUDEP after cortical seizures is SD invading the brainstem, where critical cardiovascular and respiratory control nuclei are located, effectively stopping the function of brainstem nuclei.^8,9^ Nonetheless, it remains unclear what anatomical pathways mediate the propagation of SD to the brainstem and whether specific brain activity patterns, such as seizures in particular areas, are necessary for SD to migrate away from cortical to deeper brain structures.

Previous work showed that mice expressing the S218L missense mutation in the α_1A_ subunit of Ca_v_2.1 (P/Q-type) Ca^2+^ channels (Cacna1a^S218L^), die when cortex electrically triggered seizures induce SD, which in turn spreads to the brainstem, while these mice survive when there is no SD brainstem propagation.^9^ During seizures that cause death, the superior colliculus was also invaded by SD before propagating to the brainstem. Therefore, Cain *et al*. now test whether stimulation of the superior colliculus in Cacna1a^S218L^ mice could itself induce activity leading to death. Indeed, the group found that direct optogenetic activation of the superior colliculus induced seizures, SD and death in a majority of animals with a single stimulation while all animals succumbed to sudden death at most with two subsequent stimuli. Wild-type animals did not succumb after superior colliculus stimulation.

Cain *et al*. then take advantage of an innovative diffusion-weighted MRI technique, established by the group to study migraine,^10^ to image SD in real-time in three-dimensional space by tracking water influx into cells as they swell. Wild-type mice had SD that were limited to cortical spread. However, SD in Cacna1a^S218L^ spread throughout the cortex after superior colliculus stimulation, then re-invaded the colliculi and subsequently spread to the brainstem when animals died. Similar stimulation of the thalamus caused SD that spread through cortex but did not kill the Cacna1a^S218L^ mice. Finally, Cain *et al*. used *in vitro* brain slice recordings to show that neurons in the superior colliculus of Cacna1a^S218L^ have an increased number of action potentials in response to an equivalent depolarization stimulation when compared with wild-type mice, suggesting that this increased propensity to depolarize may underly the powerful SD wave that spreads to the brainstem and leads to death. It will be critical to determine whether similar mechanisms may be at play in other genetic models of SUDEP.

In contrast, Gu *et al*. focus their study on local field potentials signals from the motor cortex and the dorsal raphe nucleus in the brainstem in the moments preceding death. They utilize mouse strains from the genetically diverse Collaborative Cross population, which come from populations bred to diversify and enlarge the genetic background of animals and screen for specific phenotypes. They previously identified four strains in which approximately half of the animals exposed to flurothyl gas, a standard model of generalized acute seizures, went on to perish from sudden death.^11^ Interestingly, as SUDEP may be confused with sudden cardiac death due to shared similar features, the authors first screen the strains for their basic heart function and find that all but one had abnormal cardiovascular function. Therefore, in subsequent experiments, they focus on the CC08 strain, which has unaffected cardiac function. Nonetheless, these results highlight an important confound in SUDEP research in that certain genetic modifications that affect the brain, may also directly modify heart function itself.

Gu *et al*. then find that CC08 mice survive flurothyl induced seizures in which oscillatory activity is recorded simultaneously in both the motor cortex and the brainstem, whereas animals paradoxically die when seizure oscillatory activity in the brainstem is absent. This potentially suggests that infra-slow activity such as SD may be present at this time in the brainstem and therefore cortical seizures transition into SD and propagate afterwards to subcortical areas causing functional arrest and then death.

A previous SUDEP clinical case study found that phase-amplitude coupling of delta (0.5–4 Hz) oscillations and high-frequency gamma oscillations (>30 Hz) in the frontal cortex disappeared when a patient died during a seizure, which is a feature normally present in data from survivors.^12^ Gu *et al*. find an overall strong delta to gamma (30–200 Hz) phase-amplitude coupling in both motor cortex and brainstem in seizures regardless of whether animals survive or not. Strikingly, phase-amplitude coupling in the motor cortex in the delta to low gamma and delta to high gamma bands is significantly reduced when animals died compared with when they survived. However, brain stem phase coupling is not reduced preceding death, suggesting that oscillatory rhythms in the motor cortex are involved in a transition to seizures becoming lethal.

Furthermore, the authors find that the level of synchrony, measured by phase-locking, is increased in the delta range during fatal seizures. Similarly, by calculating Granger causality between the motor cortex and the brainstem, Gu *et al*. find that there is a 3-fold increase in alpha (9–12 Hz), beta and gamma band connectivity in the brainstem to motor cortex direction, and an increase in gamma connectivity in the motor cortex to brainstem direction in the gamma range in seizures leading to sudden death. Overall, changes in phase-amplitude coupling, synchronization and connectivity suggest that there are specific network dynamics that enable SUDEP. It may be that particular local field potential patterns in multiple brain areas are required for a transition to SD, and its propagation to the brainstem.

Both of these studies provide intriguing new clues into the network mechanisms leading to SUDEP. SD is very likely to be the final step before cessation of the respiratory and cardiac function. However, what neuronal activity enables SD to propagate to the brainstem needs to be investigated further. It also becomes clear that multi-modal experimental methods are necessary to study these phenomena in order to understand the complex relationships between genetics, cellular activity, anatomical networks, population field potentials and SD.

## Data Availability

Data sharing is not applicable to this article as no new data were created or analyzed.

## References

[1] Devinsky O , Hesdorffer DC, Thurman DJ, Lhatoo S, Richerson G. Sudden unexpected death in epilepsy: Epidemiology, mechanisms, and prevention. Lancet Neurol 2016;15(10):1075–1088. 27571159 10.1016/S1474-4422(16)30158-2

[2] Ryvlin P , Rehims S, Lhatoo SD. Risks and predictive biomarkers of SUDEP. Curr Opin Neurol 2019;32(2):205–212. 30694923 10.1097/WCO.0000000000000668 PMC6779136

[3] Cain MS , Bernier L-P, Zhang Y, et al Hyperexcitable superior colliculus and fatal brainstem spreading depolarization in a model of sudden unexpected death in epilepsy. Brain Commun 2022:fcac006. 10.1093/braincomms/fcac006 PMC9035526 35474853

[4] Gu B , Levine NG, Xu W, Lynch RM, Pardo Manuel de Villena F, Philpot BD. Ictal neural oscillatory alterations precede sudden unexpected death in epilepsy. Brain Commun 2022:fcac073. 10.1093/braincomms/fcac073 PMC9035525 35474855

[5] Dreier JP . The role of spreading depression, spreading depolarization and spreading ischemia in neurological disease. Nat Med 2011;17(4):439–447. 21475241 10.1038/nm.2333

[6] Wei Y , Ullah G, Schiff SJ. Unification of neuronal spikes, seizures, and spreading depression. J Neurosci 2014;34(35):11733–11743. 25164668 10.1523/JNEUROSCI.0516-14.2014 PMC4145176

[7] Ullah G , Wei Y, Dahlem MA, Wechselberger M, Schiff SJ. The role of cell volume in the dynamics of seizure, spreading depression, and anoxic depolarization. PLoS Comput Biol 2015;11(8):e1004414. 26273829 10.1371/journal.pcbi.1004414 PMC4537206

[8] Aiba I , Noebels JL. Spreading depolarization in the brainstem mediates sudden cardiorespiratory arrest in mouse SUDEP models. Sci Transl Med 2015;7(282):282ra46. 10.1126/scitranslmed.aaa4050 PMC4852131 25855492

[9] Loonen ICM , Jansen NA, Cain SM, et al Brainstem spreading depolarization and cortical dynamics during fatal seizures in Cacna1a S218L mice. Brain 2019;142(2):412–425. 30649209 10.1093/brain/awy325 PMC6351775

[10] Cain SM , Bohnet B, LeDue J, et al In vivo imaging reveals that pregabalin inhibits cortical spreading depression and propagation to subcortical brain structures. Proc Natl Acad Sci U S A 2017;114(9):2401–2406. 28223480 10.1073/pnas.1614447114 PMC5338525

[11] Gu B , Shorter JR, Williams LH, et al Collaborative Cross mice reveal extreme epilepsy phenotypes and genetic loci for seizure susceptibility. Epilepsia 2020;61(9):2010–2021. 32852103 10.1111/epi.16617 PMC8011945

[12] Grigorovsky V , Jacobs D, Breton VL, et al Delta-gamma phase-amplitude coupling as a biomarker of postictal generalized EEG suppression. Brain Commun 2020;2(2):1–16. 10.1093/braincomms/fcaa182 PMC7750942 33376988

